# Co-Occurrence of Severe Equine Asthma and Palatal Disorders in Privately Owned Pleasure Horses

**DOI:** 10.3390/ani13121962

**Published:** 2023-06-12

**Authors:** Natalia Kozłowska, Małgorzata Wierzbicka, Bartosz Pawliński, Małgorzata Domino

**Affiliations:** Department of Large Animal Diseases and Clinic, Institute of Veterinary Medicine, Warsaw University of Life Sciences, 02-787 Warsaw, Poland; malgorzata_wierzbicka@sggw.edu.pl (M.W.); bartosz_pawlinski@sggw.edu.pl (B.P.)

**Keywords:** equine asthma, palatal instability, dorsal displacement of soft palate, airway diseases, equids

## Abstract

**Simple Summary:**

Asthma is one of the leading problems affecting the lower airways in equids. At the same time, palatal disorders, including primary palatal instability and dorsal displacement of the soft palate, are common problems affecting the equine upper airways. In human medicine, “one airway, one disease” is a widely accepted concept describing the anatomical, physiological, and immunological connections between upper and lower airways. Clinical studies have already highlighted co-occurrences and associations between lower airway diseases, such as asthma, and upper airway diseases, such as rhinitis, rhinosinusitis, vocal cord dysfunction, or obstructing sleep apnea in people. However, only a few studies have explored co-occurrences and associations between asthma and palatal disorder in horses. This study aimed to investigate the occurrence of palatal disorders in horses affected by severe asthma in exacerbation and to assess whether a decrease can be observed after treatment-induced disease remission. The presence of palatal disorders was investigated by overground endoscopy in forty-six horses diagnosed with severe equine asthma before and after asthma treatment. Palatal disorders were diagnosed less frequently in remission after asthma treatment than during exacerbation before treatment, suggesting that both asthma and palatal disorders may respond to similar treatment plans.

**Abstract:**

According to the “unified or united airway disease” theory, diseases in the upper and lower airways frequently co-occur because they represent a single morphological and functional unit. Palatal disorders (PDs) and severe equine asthma (SEA) are frequent diseases that, respectively, affect upper and lower equine airways; however, clinical studies focusing on the co-occurrence of PDs and SEA are limited. The present study investigated the prevalence of PDs in horses affected by SEA, and whether prevalence decreased after SEA treatment. Forty-six privately owned horses affected by SEA in exacerbation were included. For each horse, the severity of the asthma clinical signs was assessed using a previously described scoring system, and the co-occurrence of palatal disorders was investigated using overground endoscopy, before and after treatment for SEA. Before treatment (in exacerbation), 67.4% of SEA-affected horses showed evidence of PDs, including 39.1% showing evidence of palatal instability (PI) and 28.3% of dorsal displacement of the soft palate (DDSP). Airway inflammation (neutrophil percentage in the tracheal wash and bronchoalveolar lavage fluid) was worse in horses with co-occurring PDs. After treatment (in remission), no horses showed evidence of PI, while DDSP was diagnosed in 8.7% of horses. These findings suggest that palatal disorders respond to asthma treatment, supporting the hypothesis that both diseases could be manifestation of a common underlying disorder.

## 1. Introduction

Equine asthma (EA) is a disorder characterized by mild to severe chronic lower airway inflammation affecting horses of all ages and types. This disease represents a significant cause of poor performance, quality-of-life impairment, and an increase in the costs of veterinary healthcare in the equine industry [1]. The etiology is still under investigation, as the disease results from a complex interaction between genetic and environmental factors [2]. In addition to chronic airway inflammation, reversible bronchospasm and progressive airway remodeling represent pathophysiological hallmarks of the disease. The most severe form (severe equine asthma, SEA), which affects approximately 14–17% of the horse population, is clinically characterized by recurrent episodes of evident expiratory dyspnea, alternating with periods of disease remission [1,3]. The disease can be presumptively diagnosed based on history and physical examination. However, definitive confirmation requires a demonstration of ongoing lower airway obstruction through pulmonary function testing (PFT), and ongoing lower airway inflammation through airway endoscopy and broncho-alveolar lavage fluid (BALf) cytology [2,4]. While the use of endoscopy and BALf cytology is a widespread practice, PFT use remains mostly limited to research centers and is not available to several institutions [5]. Severe equine asthma is an incurable disease; however, long-term therapeutic management can help to reduce the frequency of clinical episode relapses. Bronchodilator and corticosteroid medications are used to effectively control symptoms during clinical episodes. However, successful long-term treatment strategies require implementation of various environmental interventions that aim to reduce exposure to dust and other airborne exacerbating factors [6,7].

Upper airway disorders are also common in the equine population, and include palatal disorders (PD) such as palatal instability (PI) and dorsal displacement of the soft palate (DDSP). The highest PDs prevalence occurs in the overall population of Thoroughbred and Standardbred racehorses. PDs are also common in horses working with a flexed neck, such as dressage horses and Saddlebreds [8,9]. Although a high correlation between the prevalence of PI and DDSP has been reported, it should be observed that PI often progresses to DDSP, while DDSP may occur in the absence of PI [8]. Possible etiological contributing factors include nerve (pharyngeal branch of the vagus nerve, hypoglossal nerve) or thyrohyoid muscle dysfunction [10,11,12] and congenital or acquired anatomical aberrations (epiglottic malformation, cysts, masses) [13]. Moreover, some clinical evidence suggests that local inflammation of the nasopharyngeal region may predispose individuals to obstructive upper airway diseases, such as nasopharyngeal collapse, aryepiglottic fold collapse, and dorsal displacement of the soft palate [10,13,14,15]. Clinical signs of PDs include poor athletic performance, abnormal respiratory noise, and coughing during exercise, and definite diagnosis is based on dynamic endoscopic examination (overground or high-speed treadmill endoscopy) [14,16,17,18].

In human medicine, the concept of “Unified or United Airway Disease” (UAD) or “One Airway, One Disease” has recently acquired significant clinical importance in the diagnostic and therapeutic approaches to asthma [19,20]. The evidence of co-occurring asthma and upper airway diseases, including rhinitis [21], chronic rhinosinusitis [22], obstructive sleep apnea [23], and vocal cord dysfunction [24], supports the hypothesis that common pathophysiological factors could underlie both disease processes, requiring integrated diagnostic and treatment approaches. Epidemiological evidence supports the finding that approximately 80% of asthmatics had rhinitis, and roughly 30% of patients with rhinitis had asthma [25]. One may note that in humans, the inflammatory infiltrates in the nasal and bronchial mucosa have a similar cellular composition. Thus, similar mechanisms involved in allergy development related to anatomical continuity may be suggested. Gaga et al. found, in nasal mucosa biopsies of asthmatic people, the presence of cellular infiltration even in the absence of signs of rhinitis [26]. This finding supports the hypothesis that HA and rhinitis are clinical expressions of the same disease. Moreover, in asthmatic people, a high prevalence of obstructive sleep apnea was found, caused by the permanent mucosal inflammation of the soft palate [23,26]. Even though human asthma and EA share several clinical and pathophysiological similarities, the UAD concept in equine medicine has not been thoroughly explored [3,27]. Interestingly, recent research by Joó et al. [28] has highlighted a high prevalence of DDSP in horses with EA, and particularly with SEA in exacerbation. Further investigation is worthwhile, considering the high clinical impact that both SEA and PDs have on equine health, and in particular there is a need to evaluate which factors are associated with an increased or decreased prevalence of PDs in asthmatic horses.

We hypothesized that PDs are frequent in horses with SEA exacerbation and associated with more severe clinical symptoms, and that the effective treatment of SEA leads to clinical improvement in both SEA and PDs, while an insufficient clinical response results in persisting PDs. Thus, the present study objectives are: (1) to assess the prevalence of PDs in a group of pleasure horses affected by SEA in exacerbation, and investigate whether a decrease can be observed after treatment-induced disease remission; (2) to explore whether the co-occurrence of PDs is associated with more severe asthma symptoms and/or more severe airway inflammation; and (3) to determine whether persisting PDs are associated with a decreased response to asthma treatment.

## 2. Materials and Methods

### 2.1. Study Design

A prospective, longitudinal, observational study was conducted on forty-six (*n* = 46) horses affected by SEA. Examinations were performed between 2020 and 2022 by field services from the Large Animal Veterinary Teaching Hospital, Warsaw University of Life Sciences. Initial inclusion criteria were based on a documented history consistent with SEA (recurrent episodes of respiratory distress) and the presence of an ongoing SEA exacerbation episode at the moment of baseline examination. Horses a with recent history (within the previous four weeks) of corticosteroids, bronchodilators, antibiotics, and allergen immunotherapy administration were excluded from the study. All horses underwent an initial examination (baseline) that included the collection of medical history, general physical, and detailed respiratory tract examinations, resting upper and lower airway endoscopy, tracheal wash (TW) cytology and bacterial culture, and BALf cytology. A baseline overground dynamic endoscopy was also performed the next day to investigate the presence of PDs. Starting from the day after baseline examination, the horses were treated for asthma for 21 days. The horses were subsequently re-evaluated with a follow-up examination that included general physical and detailed respiratory tract examinations, and a follow-up overground endoscopy. The clinicians performing the follow-up examinations were blinded to the results obtained at baseline examination. Both baseline and follow-up endoscopic examinations were assessed by two clinicians independently. The final scores used for statistics were decided as the mean variables; however, there were no discrepancies in the assessment of endoscopic examinations by both clinicians.

All included horses represented privately owned clinical patients. Owners provided written consent for the horse’s inclusion in the study and declared that their horses were used for recreational purposes. Since all the performed procedures represented standard diagnostic tests, no ethical approval was required.

### 2.2. Diagnostic Procedures

#### 2.2.1. History and General Physical and Respiratory Tract Examinations

Histories and clinical examinations data were collected, following the relevant professional guidelines [1]. Details regarding environmental and previous medical history were obtained. Feeding regimen, housing, access to the pasture, proximity to hay and straw storages, stable ventilation, and stable cleaning were investigated. The most recent date of corticosteroids, bronchodilators, antibiotics, or specific allergen immunotherapy was recorded [29]. In addition, any history of respiratory noises or exercise intolerance was reported. General physical examination included the evaluation of rectal temperature, heart rate, respiratory rate, mucous membrane color and hydration, capillary refill time, and lymph nodes. Detailed respiratory tract examination included tracheal and thoracic auscultation. Moreover, the presence and intensity of nasal flare, presence and amount of nasal discharge, presence and frequency of cough, and degree of abdominal lift were assessed.

#### 2.2.2. Resting Endoscopy, Tracheal Wash, and Bronchoalveolar Lavage

Resting endoscopy and cytology were performed following the relevant professional guidelines [30,31]. Endoscopic examination was carried out using a flexible video bronchoscope (8 × 2000 mm; Karl Storz, Berlin, Germany) in sedated horses. The doses of the drugs were determined based on the horse’s body weight (b.wt). Horses were sedated with detomidine hydrochloride (Domosedan; Orion Corporation, Espoo, Finland; 0.01 mg/kg b.wt i.v.) and butorphanol tartrate (Torbugesic; Zoetis Polska Sp. z o.o., Warsaw, Poland; 0.01 mg/kg b.wt i.v.). The ipsilateral nasal passage, ethmoidal region, soft palate, pharynx, larynx, trachea, and tracheal septum were examined. Bilateral guttural pouch endoscopy was performed. Tracheal mucus appearance and characteristics (accumulation, color, apparent viscosity, and localization) were determined [32].

Endoscopy-guided TW and BALf collection were performed as previously described using dedicated catheters (KRUUSE, Langeskov, Denmark) [31,33]. For TW examination, 30 mL of sterile saline was instilled to facilitate sample collection from the most ventral aspect of the trachea (tracheal puddle). Then, the samples were placed in a sterile universal container for bacteriological analysis (Deltalab, Barcelona, Spain) and in EDTA tubes for cytological analysis (Becton Dickinson, Franklin Lakes, NJ, USA). For the further reduction of coughing, 20 mL of warm lidocaine solution (Lidor 20 mg/mL, Richter Pharma, Wels, Austria) was injected in the tracheal septum area.

For BALf sampling, the catheter was passed via the ventral nasal meatus until it was wedged in a bronchus. Then, five 50 mL aliquots of sterile normal saline (Baxter, Warsaw, Poland) were instilled by syringe, aspirated immediately and placed in EDTA tubes. TW and BALf aliquots were centrifuged (300× *g* for 10 min), the supernatant was decanted, and the pellet was placed on the slides to make the smear. The smears were dried, fixated using Cytofix (Samko, Warsaw, Poland) and sent to the commercial veterinary laboratory (Laboklin Laboratory, Warsaw, Poland) for cytology and bacteriology examinations. The cytology results were returned in the form of percentage (%) of neutrophils, macrophages, and lymphocytes in TW and BALf samples separately, whereas the bacteriology results were returned in the form of bacterial growth type and intensity (lack, −; mild, +; moderate, ++; severe, +++). Possible pathogenic bacteria and probable contaminants were differentiated based on Richard et al. [34]. According to these guidelines, only common pathogens were taken into account to consider bacterial infection. Moreover, when more than three types of bacteria were found, the sample was considered to be contaminated.

#### 2.2.3. Dynamic Overground Endoscopy

Dynamic overground endoscopy was performed using portable telemetric endoscopic systems (ETL 2, head mounted Equine Training Laryngoscope; Dr Fritz Endoscopes GmbH, Tuttlingen, Germany). The nose twitch was used to restrain the horse for the safe insertion of the endoscope into the nostril and positioning in the pharynx. The battery and the telemetry system were fixed on the equine’s head using a specialized halter. Horses were ridden in the outdoor area by the usual riders who were instructed to adjust the exercise intensity to the horse performance level and the severity of clinical signs of SEA and PD. The recordings were started before the onset of exercise and terminated during the recovery phase. Laryngeal and pharyngeal function, motility of the caudal portion of soft palate, and position of the caudal border of soft palate were evaluated.

### 2.3. Classification and Scoring System for Equine Asthma

The severity of clinical signs was assessed using a modification of a scoring system recently recommended for severely asthmatic horses and assigned at baseline (prior to treatment, in exacerbation) and repeated at follow up (after treatment, in remission) [35]. The severity of airway inflammation was assigned only at baseline and assessed using a scoring system that included endoscopic (mucus appearance and characteristics) [32] and BALF cytology findings (BALf neutrophil %) [33]. For scoring purposes, % cell counts other than BALf neutrophil were not considered since SEA is typically characterized by neutrophilic inflammation [31]. The scores were assigned by two independent clinicians and summarized in Table 1. 

### 2.4. Classification of Palatal Disorders

The presence and classification of PDs was assessed at baseline (in exacerbation, before treatment) and then repeated at follow-up (after treatment, in remission), by two clinicians who independently reviewed overground endoscopy video recordings to assess interobserver repeatability and reliability. For further analysis, the mode of these observations was used.

**Figure 1:**
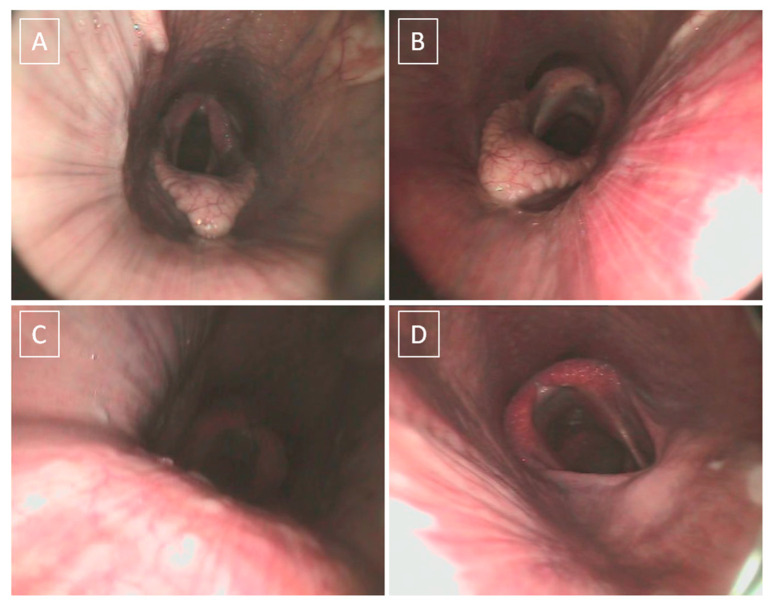
Summary of overground endoscopy findings. Normal position of the soft palate and epiglottis in a healthy horse, not included in this study (**A**). Normal position of the soft palate and epiglottis in horse with severe equine asthma (SEA). Note the hyperemia of the pharyngeal mucosa and the presence of light discharge (**B**). Palatal instability in horse with SEA. The soft palate is billowing in front of the epiglottis, obscuring the rima glottides. Although the caudal part of the pharynx cannot be visualized, dynamic assessment allows intermittent identification of the caudal border of the palate still being localized below the epiglottis (**C**). Dorsal displacement of the soft palate (DDSP) in horse with SEA. Note that the caudal edge of the soft palate is positioned dorsal to the epiglottis (**D**).

Both soft palate caudal portion motility and position were assessed, as summarized in Table 2. Palatal instability (PI) was identified when dorsoventral billowing movements of the caudal portion of the soft palate were observed, causing epiglottis flattening (Figure 1C). Dorsal displacement of the soft palate (DDSP) was diagnosed when the caudal border of the soft palate displaced above the epiglottis, preventing its visualization and obstructing the *rima glottis* (Figure 1D) [36,37].

Horses having no signs of palatal disorders were assigned the score 0, and horses showing signs of solely palatal instability or solely palatal displacement were assigned the score 1. As all horses showing DDSP signs had PI, they were assigned score 1 for DDSP (Table 2).

### 2.5. Treatment Protocols

All horses included in the study underwent a 21-day treatment period starting on the day after baseline examination, according to one of two alternative therapeutic protocols. Both treatment protocols included a combination of steroidal and bronchodilator medications, administered systemically (protocol 1) or topically via nebulization (protocol 2, Flexineb^®^, Nortev Ltd., Galway, Ireland). The protocol assigned to each horse was chosen based on clinical signs severity, owner’s preferences and/or availability of nebulization equipment. Thirty-six horses were treated according to protocol 1, while 10 horses were treated according to protocol 2. In the case of TW-positive bacterial growth, antimicrobial therapy was administered based on susceptibility results. Details of the protocols used are reported in Table 3. 

The SEA treatment protocol also included the long-term environmental change. Grass pasture and low dust feed were recommended to alleviate the clinical signs and provide long-lasting improvement [6].

### 2.6. Data Analysis

Statistical analyses were performed using the GraphPad Prism6 software (GraphPad Software Inc., San Diego, CA, USA). The level of statistical significance was set at *p* < 0.05 for all the statistical analyses performed.

#### 2.6.1. Descriptive Statistics

Descriptive statistics (percentages, number of horses) were used to analyze data collected from the history, physical examination, TW, BALf (neutrophil count), and TW bacteriology. Medians, interquartile ranges, and minimum and maximum values were calculated for ordinal variables such as clinical and mucus appearance scores, for all horses and for each group of PDs (PI, DDSP, no PDs) at baseline and follow up. Continuous variables (such as BALf and TW neutrophil %) were analyzed for normality using the Shapiro–Wilk test. Since data were not normally distributed, medians, interquartile ranges, and minimum and maximum values were calculated for all horses, and for each group of PDs.

#### 2.6.2. Prevalence of PDs

The prevalence of PDs in horses with SEA during disease exacerbation and remission were estimated. The prevalence of PI and DDSP was calculated before and after SEA treatment, separately. Expected and observed values were entered as percentages representing PI and DDSP distributions, respectively.

#### 2.6.3. Association between “Clinical Severity” Scoring, “Airway Inflammation” Scoring, and PDs Occurrence

In the dataset, each horse was assigned a “clinical severity” scoring value, an “airway inflammation” scoring value, and 0 or 1 for PI and DDSP. Horses with 1 in the PI column and 0 in the DDSP column qualified for the PI group. Horses with 1 in the PI column and 1 in the DDSP column qualified for the DDSP group. Horses with 0 in the PI column and 0 in the DDSP column qualified for the no-PD-signs group. All horses affected by both DDSP and PI were placed in the DDSP group, and thus no subject was classified as belonging to both groups. Additionally, a total group was presented as all horses’ results regardless of annotation.

The medians, interquartile range, and minimum and maximum values for the clinical scoring parameters and the % of neutrophils in airway cytology were calculated for horses without PD, PI, and DDSP, prior to asthma treatment. For clinical scoring re-evaluated after treatment, medians, interquartile ranges, and minimum and maximum values were also calculated at follow-up.

Data series % of neutrophils were tested independently for univariate distributions using a Shapiro–Wilk normality test. The data did not follow a normal distribution. For the clinical scoring parameters, the distribution was not continuous. The comparison between PI signs, DDSP signs, and the no PD signs data series was assessed using the Kruskal–Wallis test followed by the Dunn’s multiple comparisons test. As after SEA treatment no PI signs were reported, the comparison between DDSP signs and no PD signs groups was assessed using the Mann–Whitney test.

#### 2.6.4. Association between Clinical Response and PDs’ Persistence after Treatment

To compare whether the clinical scoring parameters changed after treatment, a Wilcoxon’s Signed Rank test was used. The comparison was performed only including those subjects whose classification did not change with treatment. Thus, the treatment-related comparison was performed for DDSP and no-PD signs groups only.

## 3. Results

### 3.1. Descriptive Statistics Results

The study population included 24 mares and 31 geldings of different breeds (12 mix-breeds, 3 Arabian horses, 5 Thoroughbred, 3 Wielkopolski horses, 4 Silesian horses, 1 quarter horse, 3 Hucul horses, 8 Polish Half-Bred horses, 3 Belgian horses, 2 Irish cobs and 2 Hanoverian horses), aged between 4 and 27 years (mean ± SD: 13.4 ± 5.0). All the horses presented clinical signs of SEA.

The baseline details of the horses enrolled in the study, including history, basic clinical examination, results for clinical severity scoring and airway inflammation scoring, as well as results for BALf cytology, TW cytology, and TW bacteriology are presented in the Supplementary Materials (a brief description of the history, Tables S1 and S2).

**Figure 2:**
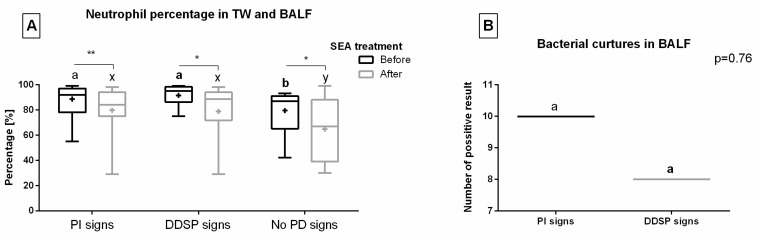
Cytology and bacteriology results obtained before severe equine asthma (SEA) treatment. Data in box plots are represented by lower quartile, median, and upper quartile, whereas whiskers represent minimum and maximum values. Additionally, the mean values are marked by “+”. Data are presented for horses with palatal instability (PI) signs, dorsal displacement of soft palate (DDSP) signs, and no palatal disorders (no PDs) signs. Neutrophil percentage in tracheal wash (TW) and broncho-alveolar lavage fluid (BALf) (**A**), and number of positive results of bacteriology in TW (**B**), are presented separately. Asterisks indicate differences between TW and BALf (* *p* < 0.05; ** *p* < 0.01). Lowercase letters indicate differences between PI, DDSP, and no PDs signs groups for *p* < 0.05.

Given that no resting endoscopy was performed after SEA treatment, the results of cytology and bacteriology were presented only before treatment. The mean neutrophil percentage in TW ranged from 79% in horses with no PDs signs to 89% and 92% in horses with PI and DDSP, respectively. Moreover, a higher neutrophil percentage in TW was found in horses with PDs than without, regardless of PD type. On the other hand, the mean neutrophil percentage in BALf ranged from 65% in horses with no PDs signs to 79% and 80% in horses with DDSP and PI, respectively. Similar to TW, a higher neutrophil percentage in BALf was found in horses with PDs than without. The neutrophil percentage in TW was higher than in BALf in total, PI signs, and DDSP signs groups, unlike the no PDs signs group (Figure 2A).

In the DDSP group, within eight TW samples, the mixed growth of at least two bacteria species was confirmed. In samples collected from four horses, a mild (+) to moderate (++) growth of *Staphylococcus aureus*, *Streptococcus equi subsp. zooepidemicus*, and *Enterobacter* spp. was found. In samples collected from two horses, a moderate (++) to severe (+++) growth of *Streptococcus equi subsp. zooepidemicus* was noted, contaminated by *Wickerhamomyces anomalus* and *Rahnella aquatilis*, respectively. In samples collected from one horse, a moderate (++) growth of *Streptococcus equi subsp. zooepidemicus* contaminated by moderate (++) growth of *Actinobacillus equuli*, *Actinobacillus rosii*, and *Enterobacter ludwigii* was demonstrated. A moderate (++) to severe (+++) growth of *Actinobacillus pleuropneumoniae*, *Lelliottia amnigena*, and *Enterobacter asburiae* was detected in the last sample, with bacterial growth in the DDSP group. In the PI group, the mixed growth of at least two bacteria species was confirmed in ten TW samples, of which eight TW were the samples described above. The remaining two TW samples showed a mild (+) to moderate (++) growth of *Streptococcus equi subsp. zooepidemicus* and *Actinobacillus pleuropneumoniae*. One of these two BALF samples also showed a mild (+) to moderate (++) growth of *Pseudomonas* spp. and *Serratia marcescens.* In the no PD signs group, no bacterial growth was found in any TW sample (Figure 2B).

### 3.2. Prevalence of PDs

During the exacerbation phase, PDs were found in 31 horses (67.4%), whereas DDSP was diagnosed in 18 horses (39.1%). One may note that in 18 horses (39.1%) the co-occurrence of PI and DDSP was noted, in 13 horses (28.3%) just PI was observed, whereas in 15 horses (32.6%) no clinical signs of PDs were recognized. After SEA treatment, in the remission phase no PI was found (0%), whereas DDSP was noted in four horses (8.7%). One may observe that in 42 horses (91.3%), no clinical signs of PDs were recognized. Comparing the pattern of PDs prevalence, PI appeared more frequently than DDSP before SEA treatment (*p* < 0.0001) as well as after SEA treatment (*p =* 0.031). Regardless of the type of diagnosed PDs, the prevalence of PI and DDSP was significantly lower after than before SEA treatment (*p* < 0.0001) (Table 4).

### 3.3. Association between “Clinical Severity” Scoring, “Airway Inflammation” Scoring, and PDs Occurrence

**Figure 3:**
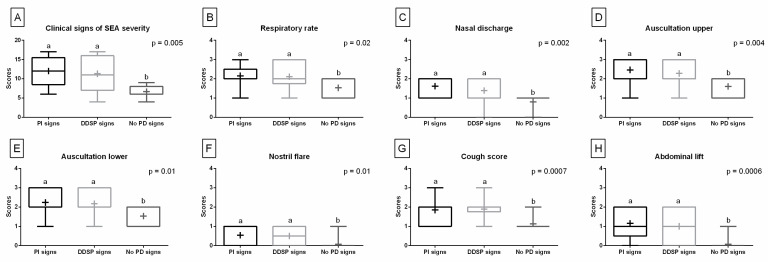
Clinical signs of the equine asthma (EA) severity quantified before treatment. Data in box plots are represented by lower quartile, median, and upper quartile, whereas whiskers represent minimum and maximum values. Additionally, the mean values are marked by “+”. Data are for horses with palatal instability (PI) signs, dorsal displacement of soft palate (DDSP) signs, and no palatal disorders (no PDs) signs. Scores are presented for the sum of the clinical signs (**A**) as well as for each of the following signs separately: respiratory rate (**B**), nasal discharge (**C**), upper respiratory tract auscultation (**D**), lower respiratory tract auscultation (**E**), nostril flare (**F**), cough score (**G**), and abdominal lift (**H**). Lowercase letters indicate differences between PI, DDSP, and no PDs signs groups for *p* < 0.05.

Comparing the quantified clinical signs obtained before SEA treatment, the scores’ values were higher in the PI and DDSP signs groups than in the no PDs signs group. No differences were found between the PI and DDSP signs groups. These differences were noted for the sum of clinical signs (Figure 3A), respiratory rate (Figure 3B), nasal discharge (Figure 3C), upper respiratory tract auscultation (Figure 3D), lower respiratory tract auscultation (Figure 3E), nostril flare (Figure 3F), cough score (Figure 3G), and abdominal lift (Figure 3H), separately.

**Figure 4:**
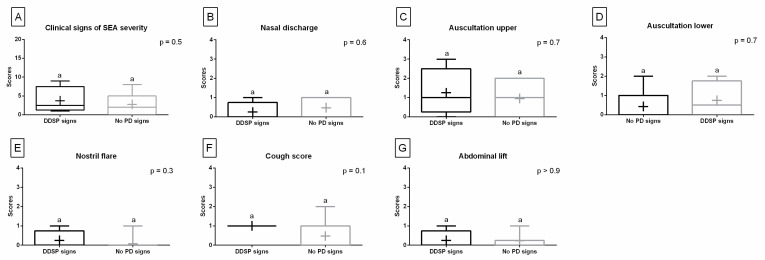
Clinical signs of the equine asthma (EA) severity quantified after treatment. Data in box plots are represented by lower quartile, median, and upper quartile, whereas whiskers represent minimum and maximum values. Additionally, the mean values are marked by “+”. Data are presented for horses with palatal instability (PI) signs, dorsal displacement of soft palate (DDSP) signs, and no palatal disorders (no PDs) signs. Scores presented for the sum of the clinical signs (**A**) as well as for each of the following signs separately: nasal discharge (**B**), upper respiratory tract auscultation (**C**), lower respiratory tract auscultation (**D**), nostril flare (**E**), cough score (**F**), and abdominal lift (**G**). Lowercase letters indicate differences between PI, DDSP, and no PDs signs groups for *p* < 0.05.

Comparing the quantified clinical signs obtained after SEA treatment, one may note that none of the examined horses showed PI signs and horses with DDSP did not show increased respiratory rate. Therefore, for these signs no bars were shown on the plots. No differences were found for the score values between the DDSP signs group and no PDs signs group for the sum of clinical signs (Figure 4A), nasal discharge (Figure 4B), upper respiratory tract auscultation (Figure 4C), lower respiratory tract auscultation (Figure 4D), nostril flare (Figure 4E), cough score (Figure 4F), and abdominal lift (Figure 4G).

### 3.4. Association between Clinical Response and PDs’ Persistence after Treatment

**Figure 5:**
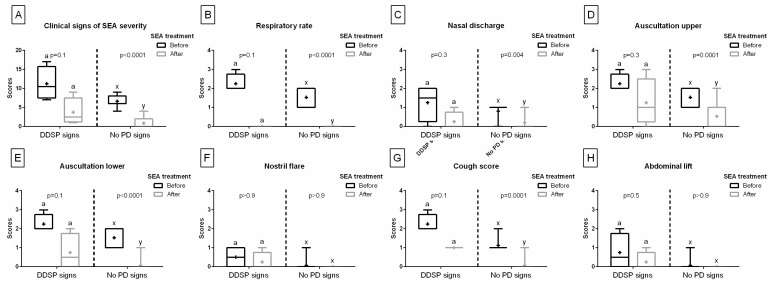
Clinical signs of the equine asthma (EA) severity compared before and after treatment. Data in box plots are represented by lower quartile, median, and upper quartile, whereas whiskers represent minimum and maximum values. Additionally, the mean values are marked by “+”. Data are presented for horses with dorsal displacement of soft palate (DDSP) signs and no palatal disorders (no PDs) signs. Scores presented for the sum of the clinical signs (**A**) as well as for each of the following signs separately: Respiratory rate (**B**), nasal discharge (**C**), upper respiratory tract auscultation (**D**), lower respiratory tract auscultation (**E**), nostril flare (**F**), cough score (**G**), and abdominal lift (**H**). Lowercase letters indicate differences between treatment periods for *p* < 0.05. As in this comparison the DDSP and No PD signs groups have not been compared, they are separated on a plot by a dashed line.

Comparing the quantified clinical signs between pre- and post-SEA-treatment sampling, one may observe a small number of horses that still showed signs of DDSP after treatment (n = 4). For these horses, no treatment-related differences were found for the sum of clinical signs (Figure 5A), respiratory rate (Figure 5B), nasal discharge (Figure 5C), upper respiratory tract auscultation (Figure 5D), lower respiratory tract auscultation (Figure 5E), nostril flare (Figure 5F), cough score (Figure 5G), and abdominal lift (Figure 5H). On the other hand, worse clinical signs in the no PDs group (n = 15) were noted before rather than after treatment for the sum of clinical signs (Figure 5A), respiratory rate (Figure 5B), nasal discharge (Figure 5C), upper respiratory tract auscultation (Figure 5D), lower respiratory tract auscultation (Figure 5E), and cough score (Figure 5G), but not for nostril flare (Figure 5F) and abdominal lift (Figure 5H).

## 4. Discussion

As equine and human asthma share many similarities, EA has been proposed as a good animal model to understand airway remodeling mechanisms and to assess the efficiency of HA treatment [3]. Thus, one may ask, may the assumptions of the UAD concept be successfully implemented in equine medicine?

Few recent research works have investigated the co-occurrence between dynamic upper airway disorders and lower airway inflammation [28,38,39,40,41,42]. Wysocka et al. found a higher prevalence of PI and nasopharyngeal collapse in various horses with mild-moderate equine asthma (mild-MEA) [39]. Courouce-Malblanc et al. evidenced the high frequency of DDSP in Trotters with lower airway inflammation [38], while Joó et al. introduced SEA as an underlying cause of DDSP [28]. The exact causality of this co-occurrence has not been proven. However, in contrast to these research works and UAD concepts stays some studies based on racehorses populations [40,41].

In our study we revealed the high prevalence of PDs in horses during SEA exacerbation, which significantly decreased after asthma treatment. Underlying causes of the PDs may be related to inflammation, as a possible cause of neuromuscular weakness of the soft palate muscles [8,43,44]. Grade 3 pharyngeal lymphoid hyperplasia (PHL) has already been associated with the occurrence of epiglottic flaccidity and DDSP [13]. Treatment regimens include anti-inflammatory steroidal or non-steroidal drug therapy. In the case of SEA, exacerbation control is based on the administration of corticosteroids and bronchodilators, systemically or topically, in association with long-term environmental changes, as relapses of clinical signs are often observed soon after drug cessation [7]. In our study all the horses showed a reduction in the signs severity of both PDs and SEA in response to steroidal administration. This may reflect that inflammation might represent an underlying component of both conditions.

One may observe that, during exacerbation, clinical signs and airway inflammation appeared more severe in horses with PDs, while these differences were not detected after treatment. Moreover, SEA horses with PDs seemed to have lower clinical responses to the treatment compared to horses without PDs. These findings show that PDs’ frequency increases with disease severity. Different possible scenarios may be proposed to explain the relationship between the presence of upper airway instability, inflammation and EA: (1) the spreading of the inflammatory process via the continuum airway epithelium, as both the upper and lower airway consist of ciliated pseudostratified columnar cells; (2) due to persistent higher negative pressure caused by increased lower airway resistance [43,44,45,46]; or (3) the independent coexistence of upper airway inflammation and/or PDs, as they were not excluded before EA exacerbation. Moreover, there was no control group used to assess the prevalence of palatal dysfunction in non-asthmatic horses, and the numbers of horses with persisting PD was low, which may have led to derived bias. Furthermore, it is worth noting that PDs create a cascade of aerodynamic problems independently in the equine respiratory tract, and even a slight soft palate elevation leads to a further increase in inspiratory negative pressure, which in turn will exacerbate the elevation [47]; thus, a more detailed experimental protocol is still required.

It has been suggested that PI always precedes the development of DDSP [36,37], suggesting that PI may represent a preliminary stage of DDSP. This is in agreement with our study, where all of the horses showing DDSP were concomitantly affected by PI. Moreover, in our study PI was more frequent than DDSP; however, after treatment only cases of DDSP were identified. In relation to the four cases with persistent DDSP after SEA treatment, we can only hypothesize the reasons behind this finding. It is possible that a permanent neuromuscular dysfunction of soft palate structures, resulted from chronic and reversible disease exacerbation or persisted from the previous coexistence of EA and DDSP. However, before EA exacerbation, none of those horses showed signs of abnormal respiratory noise or intensive cough episodes during exercise. In our caseload, anatomical malformations of the nasopharynx as a possible cause of PDs were excluded via endoscopic examination.

Another interesting fact worth considering is that exposure to systemic corticosteroids, β-2-mimetic agents, and acetylcysteine may lead to noticeable myocyte damage. In people with chronic obstructive pulmonary disease [48], as well as in horses with pulmonary dysfunction [49], structural changes in skeletal muscles were evidenced. Those alterations were considered multifactorial and were presumably caused by both the local and systemic inflammation and the administration of the above-mentioned drug therapy. Therefore, we may suggest that repeated treatments during recurrent asthma exacerbations may negatively influence the neuromuscular function of palatal muscles. However, this is only another hypothesis that needs further investigation to be confirmed or refuted.

Based on current knowledge, TW and BALf results were positively correlated with clinical signs of respiratory distress [50], whereas the association of poor performance and exercise intolerance was acknowledged only with BALf, which is the main indicator of lower airway inflammation [15,51]. In the presented caseload, one may observe higher neutrophil percentages in both BALf and TW in horses with PDs, which was in accordance with a previous study demonstrating the positive correlation between PDs’ occurrence and cytology results [38]. However, the neutrophil percentage and bacterial growth were higher in TW than BALf in SEA horses with co-occurring PDs. In contrast, the horses without PDs demonstrated similar neutrophil percentages in TW and BALf. Given that TW cytology is an indicator of tracheal inflammation, and that the trachea is directly adjacent to the upper airway, the theory of the spread of the inflammation process via the continuum airway epithelium again may be supported by the present study [14]. One may observe that a very large majority of the same bacterial communities is present in both the upper and the lower airways, showing an overlap and some continuity in the bacterial population between the two anatomical environments in healthy horses [52]. Additionally, horses with co-occurring palatal disorders had higher TW bacterial growth rate compared to asthmatic horses without palatal disorders. Weaknesses regarding bacterial culture in our study include the route of sample collection, which is anterior at avoiding contamination compared to the percutaneous transtracheal method, and secondly a low number of horses with positive culture is not sufficient to indicate an important role in etiology, having taken into account multiple independent sources of variation. However, one should note contamination as a limitation of the bacteriology sampling via endoscope.

When considering the clinical usefulness of the presented results, the following limitations of this research and future directions should be mentioned. In the current study, mild-MEA was not taken into consideration. However, one may observe that horses affected by mild-MEA are more frequently used for exercise than horses affected by SEA. Therefore, in mild-MEA horses, severe exercise intolerance is reported more frequently and the usefulness of the clinical implications of prevalence and response to the treatment of PDs in mild-MEA horses seems to be higher. Therefore, in further studies, mild-MEA horses should also be included. Responses to the SEA treatment should be considered in further research on EA and PDs, regardless of their severity. The results presented in the current study may suggest both an association between SEA and PDs and similar responses to similar treatments. Therefore, the suggested association requires further research on the dependent or independent resolution of PDs after SEA treatment, relationship, and causality. One may summarize that in horses with SEA, signs of other airway diseases are often underestimated due to focusing on the most problematic signs from the lower airways [38]. On the other hand, the neuromuscular dysfunction of the soft palate can be treated surgically to improve the athletic performance of PDs-affected horses. The recommended method of surgical treatment of DDSP is laryngeal tie-forward [8]; however, laser palatoplasty may also be performed [8]. Considering the risk of general anesthesia, surgery costs, and changeable improvement rates, a detailed respiratory tract examination, focusing not only on the upper but also on the lower airway, should be strongly recommended before surgical treatment of DDSP. According to the “UAD” concept, the detection of co-morbidities such as SEA, which may be the primary cause of PDs, can contribute to the achievement of a successful outcome with as little risk and cost of treatment as possible.

## 5. Conclusions

The current study supports the theory that inflammation might play a significant role in the pathophysiology of both diseases. In particular, co-occurring palatal disorders might reflect the presence of more widespread and severe inflammation and can contribute to diminishing the response to treatment, and facilitate complications such as lower airway bacterial contamination. One could say that conservative therapy with anti-inflammatories should always be attempted to treat palatal disorders before electing surgical intervention. Based on this study, if the horse is diagnosed only with palatal instability (no displacement) and concurrent lower airway inflammation is detected, there is a good chance that the palatal instability would resolve, after the inflammation has been treated. However, the association between SEA and PDs requires further research.

## Figures and Tables

**Table 1:** Modified score system proposed by Lavoie et al. [35] used for the quantification of the severity of variables and airway inflammation in horses affected by SEA. First seven clinical signs reflect selected results of clinical examination, whereas the subsequent five variables reflect selected results of resting endoscopy and BALf cytology, respectively [1,32]. These two types of variables are separated in Table 1 by a horizontal line. Final score is presented as a total sum of points obtained separately for variables, and endoscopic results. Final score indicates the severity of presented symptoms.

Clinical Signs	Descriptor	Score
Respiratory rate	<16	0
	17–20	1
	21–30	2
	>30	3
Nasal discharge	none	0
	serous	1
	mucopurulent	2
Tracheal auscultation	normal tracheal sounds	0
	slight increase	1
	clearly audible increased	2
	crackles and wheezing present	3
Thorax auscultation	normal pulmonary sounds	0
	slight increased pulmonary sounds	1
	clearly audible increased pulmonary sounds	2
	crackles and wheezing present	3
Nostril flare	none	0
	present	1
Cough score	none	0
	coughs at specific times of day (feeding/exercising/making beds)	1
	frequent cough	2
Abdominal lift	none	0
	slight flattening of ventral flank	1
	obvious abdominal lift and “heave line”	2
Maximum score		16
Mucus accumulation	none	0
	little, multiple small blobs	1
	moderate, larger blobs	2
	marked, confluent or stream-forming thick white to yellow	3
Mucus color	none	0
	colorless	1
	white	2
	yellow	3
Mucus localization	none	0
	½ distal	1
	proximal and distal	2
	threading	3
Mucus apparent viscosity	none	0
	fluid	1
	intermediate	2
	viscous	3
Neutrophil percentage in BALf	<5%	0
	<20%	1
	20–60%	2
	>60%	3
Maximum score		15

**Table 2:** Endoscopic characteristics of motility and position of the caudal portion of the soft palate in horses with PDs.

Endoscopic Findings	Normal (0)	Abnormal (1)
Motility of the caudal portion of soft palate	Relatively stable position, no movement or lifting present	Dorsoventral billowing movements with flattening of the epiglottis against the dorsal surface of the soft palate
Position of the caudal border of soft palate	Under the epiglottis	Above the epiglottis

**Table 3:** Details of treatment protocols used in the study.

Protocol 1				
Medication	Day	Frequency of Administration	Dosage	Route of Administration
Dexamethasone 2 mg/mL	1–3	Every 24 h	0.08 mg/kg b.wt	intramuscular
	4–6	Every 24 h	0.06 mg/kg b.wt	
	7–12	Every 48 h	0.06 mg/kg b.wt	
	13–18	Every 48 h	0.04 mg/kg b.wt	
	19–21	Every 48 h	0.02 mg/kg b.wt	
Clenbuterol 0.016 mg/g	1–14	Every 12 h	0.8 µg/kg b.wt	per os
Protocol 2				
Budesonide 500 ug/mL	1–21	Every 12 h	2000 μg/horse	nebulization
Ipratropium bromide (0.5 mg + 0.25 mg/mL)	1–14	Every 12 h	0.3 μg/kg b.wt	nebulization
Additional antimicrobial treatment				
Trimethoprim and sulfadiazine (100 mg/g + 20 mg/g)	1–7	Every 24 h	25 mg/kg b.wt	per os
Ceftiofur sodium (50 mg/mL)	1–7	Every 24 h	3 mg/kg b.wt	intramuscular

**Table 4:** The prevalence of palatal disorders (PDs) including palatal instability (PI) and dorsal displacement of the soft palate (DDSP) in horses before and after severe equine asthma (SEA) treatment.

Before SEA Treatment				
		DDSP		
		Present	Absent	Totals
PI	Present	18 (39.1%)	13 (28.3%)	31 (67.4%)
	Absent	0	15 (32.6%)	15 (32.6%)
	Totals	18 (39.1%)	28 (60.9%)	46 (100.0%)
After SEA treatment				
		DDSP		
		Present	Absent	Totals
PI	Present	0	0	0
	Absent	4 (8.7%)	42 (91.3%)	4 (8.7%)
	Totals	4 (8.7%)	42 (91.3%)	46 (100.0%)

## Data Availability

The data presented in this study are available on request from the corresponding author.

## Supplementary Materials

The following supporting information can be downloaded at: https://www.mdpi.com/article/10.3390/ani13121962/s1. A brief description of the history of horses enrolled in the study, Table S1: Details of the basic physical examination of horses enrolled in the study; Table S2: Details of the specific clinical examination of horses enrolled in the study.
