# Supplementary material for: Co-Occurrence of Severe Equine Asthma and Palatal Disorders in Privately Owned Pleasure Horses
Source: Animals (Basel). 2023 Jun 12;13(12):1962. doi: 10.3390/ani13121962 (PMC10295701; doi:10.3390/ani13121962)
Supplement: Supplementary file 1 [file animals-13-01962-s001.zip › animals-2351115-supplementary.pdf]

Supplementary Item 1:

A brief description of the history of horses enrolled in the study

Considering the feeding regimen, 80.4% of the studied horses were fed hay and concentrates, 17.4% of horses were fed soaked hay and concentrates, and 2.2% of horses were fed only hay. Among the types of housing, 73.9% of studied horses were housed in stables with boxes with access to the outside or with a window, 17.4% of horses were housed in the free-range stable, and 8.7% of horses were housed in stables with boxes with access to the corridor. Stable ventilation in 41.3% of the stables was very good, in 13.0% of the stables it was good, in 26.1% of the stables it was poor and in 2.2% of the stables, it was bad. 82.6% of the assessed stables were cleaned twice a day. Free-range stables, which accounted for 17.4% of the assessed stables, were not subject to ventilation assessment and stable cleaning. In 73.9% of the stables, hay and straw were stored in a building outside the stable, 15.2% in the stable in a separate box/storage space, and 10.9% in the stable in the attic. Given the access to the pasture, 67.4% and 32.6% of the studied horses have daily access to grassy and sandy paddocks, respectively. 84.8% of the studied horses had a history of exercise intolerance, and 15.2% did not. None of the studied horses had a history of respiratory noises and had not received corticosteroid, broncho-dilator, antibiotic, or specific allergen immunotherapy within the previous four weeks.

Table S1. Details of the basic physical examination of horses enrolled in the study. Data are expressed as mean ± SD.

**Variable**	**Baseline**			**Follow up**		
	**No PDs**	**PI only**	**DDSP with PI**	**No PDs**	**PI only**	**DDSP With PI**
**Number of horses**	15	13	18	42	0	4
Rectal temperature (ºC)	37.2 ± 0.3	37.3 ± 0.4	37.4 ± 0.4	37.2 ± 0.2	37.3 ± 0.4	37.3 ± 0.4
Heart rate (beats per minute)	45.1 ± 3.5	44.3 ± 2.6	42.3 ± 4.4	45.1 ± 3.5	44.3 ± 2.6	42.3 ± 4.4
Respiratory rate (breaths per minute)	21.2 ± 3.5	27.5 ± 6.5	28.1 ± 9.1	11.4 ± 2.5	16.0 ± 3.2	15.4 ± 2.9
Mucous membranes	normal	normal	normal	normal	normal	normal
Capillary refill time	<2s	<2s	<2s	<2s	<2s	<2s
Lymph nodes	normal	normal	Mandibular enlarged in 2 horses	normal	normal	normal

Table S2. Details of the specific clinical examination of horses enrolled in the study. The table contains: the number of horses within each group is reported, clinical severity score (median (ranges: minimum and maximum values)), airway inflammation score (median (ranges: minimum and maximum values)), BALf cytology (neutrophil %: median (ranges: minimum and maximum values)), TW cytology neutrophil % (neutrophil %,: median (ranges: minimum and maximum values)), and TW bacteriology (number of horses with positive growth).

**Variable**	**Baseline**				**Follow up**			
	**All horses**	**No PDs**	**PI only**	**DDSP with PI**	**All horses**	**No PDs**	**PI only**	**DDSP With PI**
**Number of horses**	46	15	13	18	46	42	0	4
**Total clinicalseverity score**	0-16				0-16			
Respiratory rate	2 (1-3)	2 (1-2)	2 (1-3)	2 (1-3)	0 (0-1)	0 (0-1)	n/a	0 (0-0)
Nasal discharge	1 (0-2)	1 (0-1)	2 (1-2)	2 (0-2)	0 (0-1)	1 (0-1)	n/a	0 (0-1)
Tracheal auscultation	2 (1-3)	2 (1-2)	3 (1-3)	2 (1-3)	1 (0-3)	1 (0-2)	n/a	1 (0-3)
Thorax auscultation	2 (1-3)	2 (1-2)	2 (1-3)	2 (1-3)	0 (0-1)	1 (0-1)	n/a	0.5 (0-2)
Nostril flare	0 (0-1)	0 (0)	1 (0-1)	0,5 (0-1)	0 (0-1)	0 (0-1)	n/a	0 (0-1)
Cough Score	2 (1-3)	1 (1-2)	2 (1-3)	2 (1-3)	0 (0-2)	1 (0-2)	n/a	1 (1)
Abdominal lift	0 (0-2)	0 (0-1)	1 (0-2)	1 (0-2)	0 (0-1)	0 (0-1)	n/a	0 (0-1)
**Total airway inflammation score**	0-15				n/a	n/a	n/a	n/a
Mucus accumulation	2 (0-3)	2 (0-2)	2 (1-3)	2 (1-3)	n/a	n/a	n/a	n/a
Mucus color	2 (0-3)	1 (0-2)	2 (1-3)	2 (1-3)	n/a	n/a	n/a	n/a
Mucus localization	2 (0-3)	2 (0-2)	2 (1-3)	2.5 (1-3)	n/a	n/a	n/a	n/a
Mucus apparent viscosity	2 (0-3)	2 (0-2)	2 (1-3)	2.5 (1-3)	n/a	n/a	n/a	n/a
**BALf cytology**								
Neutrophil %	81 (32-99)	70 (33-99)	82 (44-95)	89 (32-98)	n/a	n/a	n/a	n/a
**TW cytology**								
Neutrophil %	90 (42-99)	84 (42-99)	90 (55-99)	95 (75-99)	n/a	n/a	n/a	n/a
**TW bacteriology**								
Number of horses with positive growth	10	0	10	8	n/a	n/a	n/a	n/a
